# Presenting the Signers’ Eye-movements in English Reading (SEER) Corpus: An eye-tracking dataset of reading behaviors by deaf early signers and hearing non-signers

**DOI:** 10.3758/s13428-025-02881-2

**Published:** 2025-12-19

**Authors:** Frances G. Cooley, Karen Emmorey, Emily Saunders, Elizabeth R. Schotter

**Affiliations:** 1 Department of Liberal Studies, NTID, Rochester Institute of Technology, 52 Lomb Memorial Drive, Rochester, NY 14623 USA; 2 Department of Psychology, University of South Florida, 4202 East Fowler Avenue, Tampa, FL 33620 USA; 3 School of Speech, Language and Hearing Sciences, San Diego State University, 5500 Campanile Dr, San Diego, CA 92182 USA; 4 Wheelock College of Education and Human Development, Boston University, 2 Silber Way, Boston, MA 02215 USA

**Keywords:** Deafness, Reading, Corpus, Bilingualism, Eye-tracking

## Abstract

Eye-tracking corpora have advanced our understanding of reading processes by providing large-scale datasets of naturalistic reading behavior. However, existing corpora have almost exclusively sampled from typically hearing readers of spoken languages. Here, we present the Signers’ Eye-movements in English Reading (SEER) Corpus, a dataset of eye-movement behaviors from 41 skilled deaf adult readers who are early signers of American Sign Language (ASL), as well as a comparative group of 101 typically hearing monolingual English readers. Participants read 200 English sentences presented one at a time. In addition to eye-tracking data, the corpus includes detailed participant information: a standardized measure of reading proficiency, spelling recognition, and nonverbal intelligence for all participants. Information for the deaf participants include ASL comprehension scores, age of ASL acquisition, and phonological awareness scores (for a subset of participants). We report comparative analyses of reading behaviors at both the word level and sentence level. We also examine group differences in the effects of word length, frequency, and surprisal on local measures. The results indicate stronger effects of length and surprisal, but equivalent frequency effects (on content words) for deaf compared to hearing readers. The SEER Corpus offers researchers the opportunity to test hypotheses about reading development and efficiency in bimodal bilinguals who are first language users of ASL and skilled readers of English, supporting broader investigations of visual language processing. The corpus is preregistered and publicly available (https://doi.org/10.17605/OSF.IO/7P4F2) to facilitate replication, cross-study comparisons, and exploration of preliminary hypotheses in this understudied population.

## Introduction

Over decades of psycholinguistic research, scholars have sought to understand the cognitive, linguistic, and perceptual factors that contribute to language comprehension. Reading is a highly practiced, learned skill that differs fundamentally from general language acquisition, requiring years of instruction (Dehaene, 2009; Gough & Hillinger, 1980; Wolf, 2007). For deaf bimodal bilinguals, who typically acquire a sign language like American Sign Language (ASL) as their first language and English as their second language through print, reading presents a unique case. Eye tracking provides insight into the moment-to-moment mechanisms of reading that lead to behavioral decisions like saccades (rapid eye movements from one word to another) and fixations (pauses on a word or phrase). These behaviors reflect a dynamic interplay between visual, lexical, contextual, and higher-level comprehension processes, and provide a rich and multifaceted dataset. Reading behaviors are often categorized by when they occur during the time course of reading; from “early” measures, which are linked to lexical access and oculomotor planning (e.g., word skipping, first- and single-fixation duration, gaze durations), to “later” measures, which are linked to integration and reanalysis (e.g., regressions, total reading duration; Rayner, 1998, 2009; Schotter & Dillon, 2025; Staub, 2015; Staub & Rayner, 2007). In skilled reading, information is extracted not only from the word being directly fixated but also from words adjacent to fixation, enabling fluent readers to plan eye movements and anticipate upcoming content (Inhoff, 1989; Rayner, 1998; Schotter & Dillon, 2025; Schotter et al., 2012).

Most deaf adult readers learned to read in the absence of typical access to the speech sounds of their target language, making this group an interesting test case for assessing uniquely efficient reading behaviors in individuals who do not rely heavily on phonological processing (see Emmorey & Lee, 2021, for review). Despite limited access to the phonological forms of English, deaf early signers often exhibit efficient reading behaviors. In addition, deaf early signers reportedly have more efficient eye movements than matched hearing readers, including faster reading rates, shorter fixation durations, and higher skipping rates despite equivalent comprehension abilities (Bélanger et al., 2012b; Bélanger & Rayer, 2013, 2015; Cooley et al., 2025; Schotter et al., 2024; Stringer et al., 2024; Traxler et al., 2021). It is important to study deaf readers who had early access to sign language because they do not suffer from language deprivation, which leads to lifelong deficits in language fluency, academic achievement, cognitive flexibility, and underperformance on a range of linguistic and cognitive tasks (Caselli et al., 2021; Hall et al., 2019; Henner et al., 2016). As such, readers with ASL as an established first language (L1) learn to read English as a second language (L2) through print. However, although deaf bimodal bilinguals are L2 users of English, they process only one written language, making their reading experiences functionally more like L1 readers.

One possible contributor to deaf readers’ efficient reading strategies is their wider reading span, which is the spatial extent of the information they extract outside of their fixation location. In skilled hearing readers of English, the span extends 3–4 characters to the left and up to 14–15 characters to the right, depending on the particular manipulation (see Rayner, 2009, 2014; Schotter et al., 2024). However, eye-tracking studies using the moving window paradigm have shown that skilled deaf readers’ span extends beyond those of hearing readers, up to 18 characters to the right (Bélanger et al., 2012b, 2018; but see Schotter et al., 2024 for some limitations and discussion of different types of spans) and 10 characters to the left (Emmorey et al., 2025; Stringer et al., 2024). These findings are consistent with a broader literature demonstrating enhanced peripheral visual attention in deaf individuals (Dye et al., 2009), suggesting that perceptual adaptations to a lifetime of visual language use and reduced reliance on auditory input may confer advantages in how visual text is processed.

In addition to wider spans, deaf readers may also benefit from a more direct and efficient mapping between orthographic forms and semantic representations, bypassing the slower, phonology-mediated route often used by hearing readers (Bélanger et al., 2012a, 2013; Cooley & Quinto-Pozos, 2023; Costello et al., 2021; Fariña et al., 2017). While phonological awareness significantly predicts reading comprehension in hearing individuals, orthographic, semantic, and general language skills are predictive of reading comprehension in deaf signers above and beyond speech-based phonological awareness (Mayberry et al., 2011; Sehyr & Emmorey, 2022).

Building on this evidence, Bélanger and Rayner (2015) proposed the word processing efficiency hypothesis, which suggests that this direct orthographic-to-semantic pathway accounts for the fast and efficient reading behaviors observed in deaf signers. This hypothesis is supported by behavioral, event-related potential (ERP), and eye-tracking evidence indicating that phonological information plays a limited role during reading in deaf children and adult signers compared to hearing readers (Bélanger et al., 2013; Cooley & Quinto-Pozos, 2023; Costello et al., 2021; Thierfelder et al., 2020; Yan et al., 2015, 2021). Instead, orthographic and semantic processing appear to be prioritized, particularly in readers with strong ASL fluency and early sign language exposure. Importantly, deaf readers often demonstrate rapid and accurate comprehension despite skipping more words (Bélanger et al., 2012b, 2013; Cooley et al., 2025; Schotter et al., 2024; Stringer et al., 2024; Traxler et al., 2021) and spending less time fixating on them (gaze duration: Cooley et al., 2025; first fixation duration: Traxler et al., 2021). These adaptations may reflect a unique route to skilled reading shaped by visual language experience.

All of the aforementioned studies on deaf readers were designed to address one or more specific hypotheses, and involved either global visual manipulations to study the size of the spans (i.e., a moving window eye tracking paradigm; McConkie & Rayner, 1975) or manipulations of individual target words to probe the effect of a specific variable (see Rayner, 1998, 2009; Schotter & Dillon, 2025). While these studies have provided invaluable insight, they either do not necessarily reflect natural reading when the text is visually manipulated, or may have low power because the experimental manipulation of a target word is only implemented for one word on a given trial. Small sample sizes of participants and/or target words limit our opportunity to test preliminary hypotheses about how visual attention, language dominance, and cognitive sensory experiences shape reading strategies, or to explore individual differences and interactions between multiple cognitive and linguistic predictors. Therefore, a growing trend in eye-tracking research is to collect and release large datasets of eye movements during language processing, known as *corpus studies* or *megastudies*. The benefit of these types of studies is that they allow researchers to evaluate natural reading behavior, to explore the effects of new variables, and to leverage the statistical power associated with treating (almost) every word in the text as an observation (e.g., Berzak et al., 2022; Cop et al., 2017; Kliegl et al., 2004; Kuperman et al., 2023, 2024; Luke & Christianson, 2018; Nahatame et al., 2024; Siegelman et al., 2022; Sui et al., 2022).

Corpus studies have both confirmed findings from more focused empirical studies and identified findings that require high power. While there are many features of text that impact reading behaviors, three text characteristics (often referred to as the “big three”; see Kliegl et al., 2006; Rayner & Liversedge, 2011; Schotter & Dillon, 2025) have the strongest impact on whether a reader is likely to skip or fixate on a word, and the amount of time they spend fixating on it: *word length* (Blanchard et al., 1989; Brysbaert & Vitu, 1998; Rayner & McConkie, 1976; Schotter et al., 2012), *frequency* (Brysbaert & Vitu, 1998; Drieghe et al., 2008; Inhoff & Rayner, 1986; Staub & Rayner, 2007), and *predictability* (Ehrlich & Rayner, 1981; Rayner et al., 2011; Staub, 2015; Wong et al., 2024). To assess predictability effects, we calculated word *surprisal* using the large language model GPT2 (Radford et al., 2019), which indicates how surprising a word is given the entire sentence context. While other measures such as entropy or raw next-word probabilities can also be derived from language models, surprisal has been shown to align closely with processing difficulty in human reading and has a well-established theoretical foundation in the literature on L2 English reading (Berzak & Levy, 2023; Berzak et al., 2022; Hale, 2001; Levy, 2008; see Kaye & Gordon, 2025, for a review of LLM-derived predictability effects). Some of these variables may be particularly relevant for bilingual and multilingual readers, who split time reading across multiple scripts and languages (e.g., Berzak & Levy, 2023; Berzak et al., 2022; Cop et al., 2015; Whitford & Titone, 2012). For example, the less an individual is exposed to text, the lower each word’s frequency will be for each reader; as practice increases and experience is gained, frequency effects tend to diminish. Because most past studies of deaf early signers have focused on their spans or phonological decoding, few studies have investigated the effects of these variables.

However, in an analysis of manipulated target words, Cooley et al. (2025) found that deaf signers exhibited stronger effects of word length (short: 3–4 characters; long: 7–10 characters) and predictability (high cloze probability < 0.1; low cloze probability > 0.5) on skipping and gaze durations, with comparable effects of frequency (a continuous variable: log HAL [Hyperspace Analogue to Language] frequency 4.5–13.6). Based on these results, the authors argued that deaf readers are visual–linguistic processing experts, who prioritize visual information to guide their skipping behavior (i.e., because of the extremely strong length effects), but also process linguistic information in the parafovea (i.e., because predictability and frequency also impact skipping decisions). These results underscore that, despite the fact that deaf readers read in their second language, they do not experience a *frequency lag* (i.e., amplified frequency effects) that is typical of a unimodal bilingual reader (Berzak & Levy, 2023; Berzak et al., 2022; Cop et al., 2015; Gollan et al., 2011; Whitford & Titone, 2012). Comparable frequency effects to L1 readers may be due to deaf signers’ exclusive experience of reading in English, because ASL does not have a written form, making them functionally L1 readers of English (see also Emmorey et al., 2013).

### Presenting the SEER corpus

In the present study, we sought to confirm conclusions from past research about both the efficiency of deaf signers’ reading behaviors (e.g., Bélanger & Rayner, 2015; Schotter et al., 2024) and the effects of the big three variables (Cooley et al., 2025), with a large-scale corpus. The corpus includes eye-movement behaviors on every sentence, and (almost) every word within the sentence for a study that was designed to investigate controlled target words and did not involve visual manipulations or errors in text (see Cooley et al., 2025). We expected to replicate previous findings from experimental studies. For example, we expected deaf signers would read faster (i.e., more words per minute; wpm), make fewer and shorter fixations and longer saccades, and would skip more and regress less than their hearing counterparts (Bélanger et al., 2012b, 2013; Cooley et al., 2025; Schotter et al., 2024; Stringer et al., 2024; Traxler et al., 2021). In addition, we expected deaf signers to show larger effects of word length and predictability on word skipping, but equivalent effects of word frequency (Cooley et al., 2025).

In addition, we are making that dataset publicly available as the Signers’ Eye-movements in English Reading (SEER) Corpus to provide other researchers with the opportunity to investigate questions about what drives deaf signers’ eye movements. Eye-tracking data include length, frequency, and surprisal values for all words, annotations for part of speech, and a reference to the target word and its position within the sentence (see SEER Code book.csv on OSF; 10.17605/OSF.IO/7P4F2). To facilitate investigations of individual differences in addition to eye-movement measures, the dataset includes standardized scores on reading comprehension, nonverbal IQ, and spelling recognition for all readers. Additional information is provided about age of ASL acquisition, ASL comprehension, and phonological awareness scores (for a subset of deaf participants only, *n* = 15). This rich dataset will allow researchers to explore text-level and reader-level effects and to model cross-group differences in reading across modality and language background, and will help move models of reading beyond typically hearing speakers to gain broader insight into what it means to be an efficient and successful reader.

## Method

### Participants

Data were collected from 41 adult deaf signers recruited at San Diego State University (SDSU), the University of Texas at Austin (UT), Gallaudet University (GU), and the National Technical Institute for the Deaf (NTID), and 101 hearing native English speakers recruited at the University of South Florida (USF). Approval for the study was obtained from the institutional review boards at these institutions. All participants were aged between 18 and 55 years, had normal or corrected-to-normal vision, were proficient readers of English, and reported no history of speech/language or cognitive impairments. Deaf participants were prelingually and profoundly deaf, identified ASL as a primary language, and used ASL predominantly for communication with family and friends. Hearing participants were native speakers of English, had no knowledge of ASL, and reported normal hearing.

Informed consent was given in writing after deaf participants received a detailed explanation of the consent forms in ASL, delivered by either a fluent hearing signer or a native deaf signer. Compensation for participation was provided at a rate of $10 per half-hour or via course credit. All participants filled out language and demographic questionnaires. Deaf participants completed an additional questionnaire that included details about their family deafness and signing history, age of first ASL exposure, current ASL usage, and education history. Eighteen participants reported learning ASL from at least one deaf signing parent, and 12 additional participants reported learning ASL from at least one hearing signing parent. In addition, all participants completed the language and cognitive assessments listed below. Table 1 shows the language and cognitive scores for all participants, along with *t*-test *p*-values to highlight group differences for each measure.

**Table 1:** Participant age and education information, PIAT-R, KBIT, Spelling Recognition, and eye-tracking task comprehension question accuracy for all participants; ASL-CT scores, age of ASL acquisition, phonological awareness scores for deaf participants only

	Hearing; *n* = 101 Mean (*SD*) [Range]	Deaf; *n* = 41 Mean (*SD*) [Range]	*p*-value
Age (years)	26.42 (9.4) [18–51]	32.62 (7.7) [18–52]	**< 0.001**
Education (years in college)	3.5 (2.6) [0–13]	6.23 (3.2) [0–15]	**< 0.001**
PIAT-R	86.26 (9.1) [61–97]	83.36 (10.2) [65–99]	0.1
KBIT score	38.41 (3.6) [29–46]	38.31 (5.1) [23–46]	0.54
Spelling Recognition score	73.8 (7.1) [54–86]	73.1 (9.1) [47–87]	0.51
Eye-tracking task comprehension accuracy	94.1 (0.04) [75.5–98]	87.31 (0.11) [53.1–98]	**< 0.001**
Phonological awareness task^a^	–	0.635 (0.15) [0.45–0.93]	–
ASL age of acquisition	–	1.6 (2.1) [0–8]	**–**
ASL-CT	–	24.91 (3.1)^b^ [16–29]	–

mean (*SD*) [range]. Bolded *p*-values indicate significance

^a^Several participants also completed the phonological awareness task developed by Hirshorn et al. (2015) as part of other studies (*n* = 15) and thus have existing phonological awareness scores that are included in the behavioral data posted on OSF. Performance falls within the mean and *SD* reported by Sehyr and Emmorey (2022) for deaf early signers (*M* =.65; *SD* =.14) and below that for hearing readers (*M* =.88; *SD* =.11)

^b^Deaf readers only; performance falls within the mean and *SD* reported by Hauser et al. (2016) for deaf native signers (*M* = 26, *SD* = 2.3)

#### Peabody Individual Achievement Test-Revised (PIAT-R)

English reading proficiency was evaluated using the PIAT-R (Dunn & Markwardt, 1989; test–retest reliability *r* = 0.88), a standardized reading comprehension test involving matching the meanings of written sentences to one of four pictures. The test items increase in difficulty, incorporating complex vocabulary and syntax. To shorten the assessment, testing began at item 60, as adults generally perform well on earlier items. The test was discontinued when participants answered five out of seven consecutive items incorrectly.

#### Andrews spelling recognition test

Spelling proficiency was measured using the Andrews Spelling Recognition Test (Andrews & Hersch, 2010; test–retest reliability *r* = 0.93). Participants were shown 88 words and asked to identify any misspelled items by circling them. Scores were based on the number of correctly identified items, with deductions for incorrectly marked items. Due to the use of British/Australian spelling in this test, one item (“behaviour”) was not scored.

#### American Sign Language-Comprehension Test (ASL-CT)

ASL comprehension was assessed using the ASL-CT (Hauser et al., 2016; internal reliability *α* = 0.83). Participants viewed a static photo, real-life video, or ASL production, and selected the corresponding item from an array of four options. The test was designed to target complex ASL structures, with all ASL items signed by a native deaf signer. This 30-item test was administered only to deaf participants.

#### Kaufman Brief Intelligence Test (KBIT-2)

Nonverbal reasoning was evaluated using the KBIT-2 (Kaufman & Kaufman, 2004; test–retest reliability *r* = 0.85). In this task, participants were presented with an array of visual objects and asked to select the one that completed the sequence. This task concludes after all 46 items are completed or participants ceiling out after four consecutive errors.

### Stimuli and design

The eye-tracking task included 200 sentences (7–21 words; median 15 words) that had been created to investigate reading behavior on particular target words (see Cooley et al., 2025). Sentences ranged in estimated reading level and clause complexity. Two sentences were removed from this analysis due to typos. Table 2 contains word-level descriptive statistics for all words in the corpus, excluding sentence-initial, sentence-final, and target words; Table 3 contains sentence-level descriptive statistics for all sentences included in this dataset.

**Table 2:** Word-level descriptive statistics (*n* = 2,343)

	Length	Frequency	Surprisal
Mean (*SD*) [Range]	4.4 (2.2) [1–13]	12.65 (2.98) [4.19–16.96]	6.3 (4.4) [0.003–24.25]

Length reflects number of characters; frequency is log HAL frequency from the English Lexicon Project (Balota et al., 2007); surprisal was calculated by GPT2

**Table 3:** Sentence-level descriptive statistics (*n* = 198)

	Clause complexity	Grade level	Number of clauses	Number of words
Mean (*SD*) [Range]	0.42 (0.45) [0–1]	8.6 (2.1) [4–13]	1.55 (0.7) [1–4]	15.14 (2.23) [7–21]

Clause complexity was determined using the Haiyang Ai web-based L2 syntactic analyzer (Lu & Ai, 2015). Reading grade level was determined by the INK Reading Level Checker (INK Co., n.d.)

### Apparatus and recording

Eye movements were recorded using an SR Research EyeLink 1000 Plus eye tracker in a desktop setup (1,000 Hz; SDSU, USF) or an SR Research Eyelink Duo eye tracker (1,000 Hz; UT, GU). Stimuli were presented on an LCD monitor at a viewing distance of 65 cm (USF), 85 cm (SDSU), or 55 cm (UT, GU), with each character subtending 0.27, 0.22, or 0.32 degrees of visual angle, respectively, at size 14 font.^1^ To minimize movements, participants used a chin and headrest. Calibration was repeated as needed throughout the experiment. While the viewing was binocular, only movements of the right eye were recorded. Participants made manual responses on a response pad to end a trial and to respond to comprehension questions.

### Procedure

Following a drift check, participants fixated on a box on the far left of the screen. Once the box was fixated, the sentence appeared such that the first word was in the same location as the box. Text was displayed in black 14-point Courier New on a light gray background. After reading the sentence for understanding, participants looked at a sticker on the far right of the screen and pressed a button to indicate that they had completed the trial. Simple yes-or-no questions followed 25% of sentences to ensure participants were paying attention and reading for comprehension. Following five practice trials with questions after each, the task began.

## Results

### Description of corpus data

We summarize, for each group, both global reading behaviors (i.e., at the sentence level; Table 4) and local reading behaviors (i.e., at the word level; Table 5; see Schotter & Dillon, 2025), and include *p*-values for (generalized) linear mixed-effects regression models testing group differences in reading behaviors with random effects of sentence (for global measures) or word ID (for local measures) and subject. Analyses were conducted on all words, excluding sentence-first, sentence-last, and target words.

**Table 4:** Sentence-level reading behaviors for all deaf and hearing readers in the corpus

	Hearing	Deaf	*p*-value
Reading rate (words per minute)	300.9 (133.2)	365 (163.4)	** < 0.01**
Trial fixation count	12.65 (4.6)	11.22 (4.4)	** < 0.05**
Mean fixation duration (ms)	246.33 (106.5)	233.72 (100.2)	** < 0.01**
Mean saccade length	8.38 (8.3)	9.58 (9.4)	** < 0.001**
Percent regressions	21.64 (41)	17.63 (37.7)	** < 0.001**

Mean (*SD*)

**Table 5:** Local reading behaviors for all deaf and hearing readers in the corpus

	Hearing (*n* = 101)	Deaf (*n* = 41)	*p*-value
Skipping	0.42 (0.5)	0.49 (0.5)	** < 0.01**
Launch site	7.93 (10.5)	8.9 (12)	** < 0.05**
Landing position	4.37 (2.9)	4.57 (3.1)	** < 0.01**
Single fixation duration (ms)	227.56 (81.8)	213.98 (77.74)	** < 0.05**
First fixation duration (ms)	226.97 (85.7)	216.74 (80.1)	** < 0.05**
Gaze duration (ms)	246.14 (109.2)	233.59 (104.2)	** < 0.05**
Total duration (ms)	294.56 (238.7)	274.77 (252.6)	0.08^✝^
Regressions in	0.14 (0.4)	0.12 (0.2)	** < 0.05**
Regressions out	0.09 (0.3)	0.06 (0.2)	** < 0.001**
Refixation probability	0.12 (0.3)	0.09 (0.3)	** < 0.05**
Rereading probability	0.06 (0.2)	0.04 (0.2)	** < 0.001**

Mean (*SD*); bolded *p*-values indicate significance; ^✝^marginally significant (*p* = 0.08)

Global measures included *reading rate* (i.e., the average words per minute), *trial fixation count* (i.e., the total number of fixations per sentence), *mean fixation duration* (i.e., the average of all fixations), *mean saccade length* (i.e., the average number of characters between fixations), and *percent regressions* (i.e., the average number of regressions per sentence). Each of these measures differed significantly between the groups, with deaf signers exhibiting eye movement patterns that indicate more efficient reading.

Local reading measurements included *skipping probability* (i.e., whether the reader skipped or fixated the word during first pass), *launch site* (i.e., the number of characters into a word that a reader will trigger a saccade to the next word), *landing position* (i.e., the number of characters from the beginning of a word where the reader’s eyes first land), *percent skipping* (i.e., the average number of words skipped in a given sentence), *single fixation duration* (i.e., the length of first fixation if only one fixation is made on the word), *first fixation duration* (i.e., the amount of time [ms] spent during the first fixation prior to moving the eyes), *gaze duration* (i.e., the cumulative fixation durations on the word prior to leaving the word), *refixation probability* (i.e., the proportion of words that have more than one first-pass fixation), *regressions in* (i.e., the number of regressive saccades made to each word if it had been read during first pass), *regressions out* (i.e., the number of regressive saccades triggered by each word), *rereading probability* (i.e., the number of regressive saccades back to words that had previously been fixated on), and *total duration* (i.e., the total amount of time spent reading the word during first, second etc. passes). There were significant differences between the groups for all these measures, except for total reading time, which was marginally significant (faster for deaf signers; *p* = 0.061).

#### Interindividual consistency of reading duration

Eye movements during reading vary widely between individuals. To assess the reliability of each effect we calculated the split-half correlations for the local (Table 6) and global reading measures (Table 7) separately for each group.

**Table 6:** Spearman–Brown split-half reliability coefficients for global reading measures in the SEER Corpus

	Hearing	Deaf
Reading rate	0.92	0.93
Trial fixation count	0.89	0.89
Mean fixation duration	0.78	0.80
Mean saccade length	0.62	0.62
Percent regressions	0.75	0.51
Percent skipping	0.92	0.95

**Table 7:** Spearman–Brown split-half reliability coefficients for local reading measures in the SEER Corpus

	Hearing	Deaf
First fixation duration	0.61	0.54
Single fixation duration	0.63	0.47
Gaze duration	0.73	0.70
Total reading duration	0.69	0.54
Landing position	0.87	0.88
Launch site	0.43	0.47
Skip rate	0.88	0.90
Regressions in	0.67	0.56
Regressions out	0.51	0.39
Rereading probability	0.63	0.53

#### Analysis 1: Text characteristic effects on all words in the corpus

**Fig. 1:**
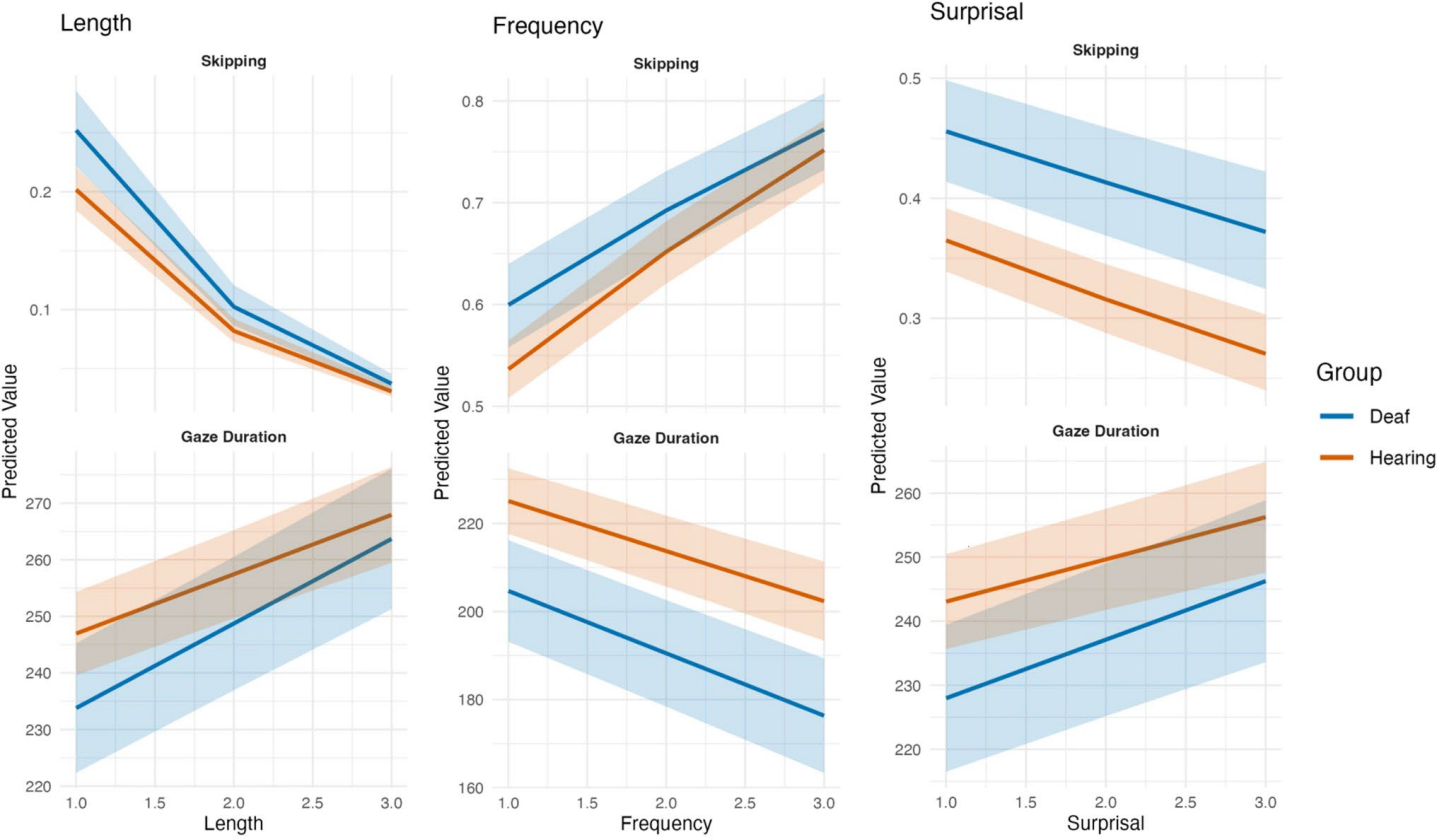
Skipping and gaze durations by length, frequency, and surprisal for deaf and hearing readers. *Note*: Predicted values are plotted separately for deaf and hearing readers across standardized values (–2 to + 2 *SD*) of each predictor: length, frequency, and surprisal. The *y*-axis shows model-estimated values for the dependent reading measure; shaded regions indicate 95% confidence intervals

To assess word characteristic effects, we analyzed the local measures with (generalized) linear mixed effects regression models with predictors for participant group (deaf vs. hearing), length, frequency, and surprisal, and the interactions between group and the lexical variables with random effects of word and subject (Tables 8 and 9). These analyses were preregistered on OSF. For fixation duration data, we used linear models, and for binary measures, we used generalized glmer with family set to binomial via the lme4 package in RStudio (Bates et al., 2015). All continuous predictors were mean-centered and scaled. Group was contrast-coded using sum-to-zero coding, so that the main effects of the lexical variables represent the effects averaged across group, and the main effect of group represents how the deaf readers compare to the hearing readers. When assessing the interactions, if the interaction is significant and the effect estimate has the same sign as the main effect (i.e., both positive or both negative), it indicates that the effect is stronger for deaf readers. In contrast, when the signs of the main effect and the interaction term differ (i.e., one is positive and the other is negative), the effect is weaker for deaf readers. See Fig. 1 for a visualization of predicted skipping and gaze duration values for length, frequency, and surprisal to see how interaction terms impact the magnitude of effects.

**Table 8:** Analysis of length, frequency, and surprisal effects for deaf and hearing readers for early measures

Predictors	Skipping				Refixations				Single fixation duration				First fixation duration				Gaze duration			
	Est	SE	t/z	p	Est	SE	t/z	p	Est	SE	t/z	p	Est	SE	t/z	p	Est	SE	t/z	p
(Intercept)	0.83	0.04	−3.56	** < 0.001**	0.05	0.01	−28.19	** < 0.001**	218.31	2.87	76.00	** < 0.001**	215.88	2.69	80.19	** < 0.001**	227.35	3.45	65.99	** < 0.001**
Length (L)	0.34	0.01	−56.38	** < 0.001**	2.26	0.05	34.96	** < 0.001**	2.91	0.61	4.76	** < 0.001**	2.53	0.54	4.72	** < 0.001**	12.73	0.74	17.13	** < 0.001**
Frequency (F)	1.55	0.03	21.04	** < 0.001**	0.65	0.02	−15.79	** < 0.001**	−9.73	0.72	−13.51	** < 0.001**	−8.68	0.64	−13.63	** < 0.001**	−12.52	0.88	−14.18	** < 0.001**
Surprisal (S)	0.81	0.02	−10.60	** < 0.001**	1.40	0.04	13.56	** < 0.001**	3.92	0.65	5.98	** < 0.001**	2.84	0.57	4.95	** < 0.001**	7.78	0.80	9.76	** < 0.001**
Group (G)	1.39	0.14	3.34	**0.001**	0.52	0.11	−3.05	**0.002**	−14.97	5.70	−2.62	**0.009**	−14.99	5.35	−2.80	**0.005**	−17.35	6.84	−2.54	**0.011**
L * G	0.96	0.02	−2.44	**0.015**	1.12	0.02	5.79	** < 0.001**	4.59	0.64	7.17	** < 0.001**	3.94	0.58	6.79	** < 0.001**	4.42	0.75	5.87	** < 0.001**
F * G	0.92	0.02	−4.61	** < 0.001**	0.88	0.02	−4.85	** < 0.001**	−2.50	0.79	−3.18	**0.001**	−1.94	0.72	−2.69	**0.007**	−2.72	0.95	−2.87	**0.004**
S * G	1.04	0.02	2.56	**0.011**	1.03	0.02	1.20	0.231	2.19	0.68	3.20	**0.001**	1.72	0.62	2.77	**0.006**	2.47	0.81	3.04	**0.002**
Random Effects																				
σ^2^	3.29				3.29				5231.82				6013.74				9518.80			
τ_00_	0.29_word_id_				0.41_word_id_				262.46_word_id_				194.30_word_id_				390.67_word_id_			
	0.29_SUBJECT_				1.32_SUBJECT_				942.68_SUBJECT_				830.00_SUBJECT_				1354.73_SUBJECT_			
ICC	0.15				0.34				0.19				0.15				0.15			
N	142_SUBJECT_				142_SUBJECT_				142_SUBJECT_				142_SUBJECT_				142_SUBJECT_			
	2486_word_id_				2486_word_id_				2421_word_id_				2428_word_id_				2426_word_id_			
Observations	313,065				313,065				140,468				191,326				174,324			
Marginal R^2^/Conditional R^2^	0.234/0.348				0.156/0.447				0.017/0.201				0.013/0.157				0.032/0.182

**Table 9:** Analysis of length, frequency, and surprisal effects for deaf and hearing readers for late measures

Predictors	Regressions out				Regressions in				Rereading probability				Total duration			
	*Est*	*SE*	*t/z*	*p*	*Est*	*SE*	*t/z*	*p*	*Est*	*SE*	*t/z*	*p*	*Est*	*SE*	*t/z*	*p*
(Intercept)	0.05	0.00	−36.57	** < 0.001**	0.09	0.01	−27.62	** < 0.001**	0.03	0.00	−39.03	** < 0.001**	270.97	6.34	42.77	** < 0.001**
Length (L)	0.95	0.02	−2.13	**0.033**	0.75	0.02	−11.82	** < 0.001**	1.24	0.03	7.77	** < 0.001**	11.27	2.01	5.60	** < 0.001**
Frequency (F)	1.00	0.03	0.00	0.998	1.06	0.03	2.09	**0.037**	0.82	0.03	−6.04	** < 0.001**	−11.93	2.37	−5.03	** < 0.001**
Surprisal (S)	1.07	0.03	2.46	**0.014**	1.31	0.03	10.61	** < 0.001**	1.06	0.03	1.85	0.065	8.22	2.17	3.79	** < 0.001**
Group (G)	0.57	0.10	−3.35	**0.001**	0.65	0.11	−2.56	**0.011**	0.53	0.10	−3.53	** < 0.001**	−24.79	12.44	−1.99	**0.046**
L * G	0.93	0.03	−2.53	**0.011**	0.97	0.02	−1.40	0.162	0.99	0.03	−0.41	0.685	3.75	1.74	2.15	**0.032**
F * G	1.05	0.04	1.23	0.217	0.80	0.02	−7.23	** < 0.001**	1.00	0.03	−0.14	0.892	−4.92	2.20	−2.24	**0.025**
S * G	1.05	0.03	1.43	0.153	1.04	0.03	1.58	0.115	1.02	0.03	0.80	0.425	2.26	1.88	1.20	0.230
Random Effects																
σ^2^	3.29				3.29				3.29				50,963.92			
τ_00_	0.35_word_id_				0.39_word_id_				0.53_word_id_				3237.64_word_id_			
ICC	0.77_SUBJECT_				0.83_SUBJECT_				0.91 _SUBJECT_				4463.40 _SUBJECT_			
N	0.25				0.27				0.31				0.13			
	142_SUBJECT_				142_SUBJECT_				142 _SUBJECT_				142 _SUBJECT_			
	2428_word id_				2428_word_id_				2486 _word id_				2426 _word_id_			
Observations	191,326				191,326				313,065				174,324			
Marginal R^2^/Conditional R^2^	0.016/0.266				0.022/0.286				0.033/0.328				0.006/0.137

$$\mathrm{Measurement}\hspace{0.17em}\sim \hspace{0.17em}\left(\mathrm{centered}\_\mathrm{length}+\mathrm{centered}\_\mathrm{surprisal}+\mathrm{centered}\_\mathrm{frequency}\right)*\mathrm{Group}+\left(1\left|\mathrm{SUBJECT}\right.\right)+\left(1\left|\mathrm{WORD}\right.\right).$$

#### Length effects

Word length affected all eye-movement measures (all *p*s < 0.001). Longer words were less likely to be skipped, triggered more refixations, were more likely to be reread, and incurred longer fixation durations than shorter words. Deaf readers had stronger length effects than hearing readers for all eye-movement measurements (early measures: all *p*s < 0.001; late measures: *p*s < 0.05), with the exception of equivalent length effects on regressions in and rereading probability.

#### Frequency effects

Frequency effects affected all eye-movement measurements (all *p*s < 0.01) with the exception of regressions out. High-frequency words were more likely to be skipped and had shorter fixation durations. Deaf readers had weaker frequency effects on skipping and regressions in, but *stronger* frequency effects on refixations and fixation durations than hearing readers.

#### Surprisal effects

Surprisal effects were significant for skipping and all early fixation duration measures (*p*s < 0.05). Deaf readers had weaker surprisal effects on skipping but stronger effects on early fixation duration measures than hearing readers.

#### Analysis 2: Analysis of content words in the corpus

The patterns of frequency observed in this corpus analysis (weaker frequency effects on skipping and stronger frequency effects on gaze durations for deaf readers compared to hearing readers) did not replicate the effects reported in the target word study of Cooley et al. (2025), who reported a null interaction between frequency and group. One key methodological difference may account for this discrepancy: the target words analyzed by Cooley et al. (2025) were content words, whereas the corpus analysis also included function words. To test whether the frequency by group interaction is not observed on content words even when including the larger dataset of the corpus, we ran an exploratory analysis of skipping and gaze durations on content words only (213,090 observations, ~ 70%; 1,476 words; Table 10).

**Table 10:** Word-level descriptive statistics for content words only (*n* = 1,476)

	Length	Frequency	Surprisal
Mean (*SD*) [Range]	5.4 (2.1) [1–13]	10.82 (2.2) [4.19–16.96]	8.12 (4.4) [0.008–24.16]

Length reflects number of characters; frequency is log HAL frequency from the English Lexicon Project (Balota et al., 2007); surprisal was calculated by GPT2

In this subset analysis, we replicated all of the effects that were similar between the larger corpus analysis above and Cooley et al. (2025); compared to hearing readers, deaf readers skipped content words more frequently (*p* < 0.001), had shorter gaze durations (*p* < 0.02), showed stronger length effects on both skipping and gaze durations (*p* < 0.001), weaker surprisal effects on skipping (*p* < 0.001), and stronger surprisal effects on gaze durations (*p* < 0.01). Most importantly, this analysis also replicated the null interaction between group and frequency reported by Cooley et al. (2025), suggesting that for content words, deaf and hearing readers are equally sensitive to word frequency (see Table 11 for model output). We address the potential difference between function and content words for deaf readers in the discussion section.

**Table 11:** Analysis of length, frequency, and surprisal effects for deaf and hearing readers for skipping and gaze duration; content words only (*n* = 1,715)

*Predictors*	Skipping, content words				Gaze duration, content words			
	*Est*	*SE*	*t/z*	*p*	*Est*	*SE*	*t/z*	*p*
(Intercept)	0.73	0.05	−5.01	** < 0.001**	227.11	3.68	61.63	** < 0.001**
Length (L)	0.32	0.01	−47.34	** < 0.001**	14.42	0.92	15.59	** < 0.001**
Frequency (F)	1.25	0.04	7.22	** < 0.001**	−13.14	1.28	−10.24	** < 0.001**
Surprisal (S)	0.78	0.02	−10.42	** < 0.001**	8.04	0.97	8.27	** < 0.001**
Group (G)	1.46	0.18	3.09	**0.002**	−16.86	7.24	−2.33	**0.020**
L * G	0.89	0.02	−5.00	** < 0.001**	4.88	0.86	5.68	** < 0.001**
F * G	0.98	0.03	−0.93	0.352	−1.55	1.21	−1.28	0.201
S * G	1.09	0.02	4.55	** < 0.001**	2.45	0.92	2.68	**0.007**
Random Effects								
σ^2^	3.29				10,173.54			
τ_00_	0.31 word_id				458.61 word_id			
ICC	0.18				0.16			
N	1534 word_id				1476 word_id			
	142 SUBJECT				142 SUBJECT			
Observations	188,943				127,184			
Marginal R^2^/Conditional R^2^	0.181/0.333				0.027/0.184			

## Discussion

The SEER Corpus offers a large-scale, preregistered, openly available dataset of eye-movement behavior from deaf and hearing readers who have similar reading abilities. In addition to eye-movement data, we include reader-level measures of reading comprehension ability, spelling recognition skill, and nonverbal intelligence (for all readers) as well as ASL comprehension, deafness, and language history information for the deaf readers, and phonological awareness scores for a subset of 15 deaf participants. We are releasing this dataset to the broader scientific community with the goal of furthering our understanding of what it means to be a skilled, efficient reader.

The current analysis provides corpus-level evidence that lifelong deafness and early exposure to a visual language change the reading process. In particular, deaf early signers have significantly more efficient local- and global-level eye movements for all but one reported measure (total fixation time, *p* = 0.061). Analyses of the SEER corpus replicate and extend previous findings from experimental studies demonstrating that deaf early signers are highly efficient readers. Specifically, they have faster reading rates, skip more words, make fewer regressions and refixations, and have shorter fixation durations and longer saccades than their hearing peers (Bélanger & Rayner, 2015; Bélanger et al., 2012b, 2013; Cooley et al., 2025; Schotter et al., 2024; Stringer et al., 2024; Traxler et al., 2021). Our data reinforce that this efficiency is not exclusively found in experimental studies using unnatural visual manipulations (such as moving window or boundary paradigms), but also during relatively natural reading. The results described here provide additional validity to prior findings as well as support for the hypothesis that deaf early signers have a distinct, visually guided pathway to reading proficiency.

Corpus-level effects of length, frequency, and surprisal provide further nuance compared to target-level analyses reported by Cooley et al. (2025). In the corpus analysis, differences in sensitivity to word frequency emerged across the time course of word reading, including weaker frequency effects on skipping and stronger frequency effects on gaze duration. However, the more analogous analysis of only content words from the corpus revealed equivalent frequency effects on skipping and gaze durations for deaf and hearing readers. In English, function words (e.g., *is*, *to*, *and*) often appear as independent words to express grammatical relationships. In contrast, ASL typically conveys the same information morphologically (e.g., through inflection, facial expressions, handshape changes, or movement patterns) rather than separate signs functioning as standalone function words (Bellugi & Klima, 1979; Liddell, 2003; Supalla & Newport, 1978; Valli et al., 2011). As a result, deaf readers may engage with English function words differently due to the linguistic structure of their L1 ASL. A more in-depth analysis of function vs. content words may be an interesting avenue for future uses of the SEER corpus.

Cooley et al.’s (2025) target analysis reported cloze predictability effects, assessed by the degree to which readers predict a specific word when given the preceding sentence context on a cloze task (e.g., Taylor, 1956). In the present study, we used GPT2 to calculate each word’s *surprisal*, which is the degree to which each word is “surprising” in the context of the entire sentence, and can be considered the inverse of predictability. Surprisal effects emerged in both groups. Similar to predictability effects on target words, the stronger surprisal effects on fixation durations at the corpus level for deaf readers indicate that contextual information influences processing during fixations. Differences in surprisal (and predictability) effects between groups suggests that deaf readers may rely on in-the-moment semantic integration during fixations more so than hearing readers, rather than anticipating the meaning of upcoming words in advance.

When considering the “big three” effects together, it becomes clear that deaf readers prioritize visual information (i.e., word length) to determine which words to skip or fixate and how long to spend during fixations. Specifically, deaf readers skip short words significantly more often and fixate on long words more often than hearing readers. For deaf readers, word length plays a dominant role throughout the time course of reading, continuing to influence fixation durations and rereading decisions. This exaggerated sensitivity to the visual characteristics of words likely reflects adaptations to a lifetime of processing language visually and drawing on enhanced peripheral attention (Dye et al., 2009). However, deaf readers also exhibited strong effects of frequency and surprisal on skipping, suggesting that they access linguistic information early in the reading process, and therefore they are not solely dependent on visual cues to guide which words should be fixated. Hearing readers, in contrast, prioritize frequency and surprisal (along with word length) during skipping and rereading decisions. While both groups ultimately integrate visual, lexical, and contextual cues, they appear to do so with different timing and emphasis.

At the global sentence level, deaf readers demonstrated less rereading and fewer regressions than hearing readers. These sentence-level metrics suggest that deaf signers are capable of constructing coherent interpretations of text with minimal need for backtracking, possibly due to their enhanced ability to extract information from the parafovea. These findings provide additional support for the enhanced reading spans reported to the left and right of fixation for deaf readers (Bélanger et al., 2012a, 2012b, 2018; Emmorey et al., 2025; Liu et al., 2021; Schotter et al., 2024; Stringer et al., 2024). Their broader spans may allow them to plan which longer, content-rich words to fixate on and engage in efficient lexical retrieval and contextual integration.

The deaf and hearing readers included in this corpus are matched for overall reading proficiency, as measured by the PIAT. The eye-tracking task included yes or no comprehension questions following 40 sentences, which both groups performed very well in answering. Hearing readers answered ~ 37 questions correctly (94.1%) while deaf readers answered ~ 36 questions correctly (87.3%). Although small, the difference was statistically significant. We suggest this difference may be due to deaf readers’ bilingualism. Individuals who split time between more than one language have overall less experience in each of their target languages, resulting in differences in comprehension for monolingual and bilingual users of the same language. Indeed, similar findings have been reported in the eye-tracking literature with deaf signers. Traxler and colleagues (2021) reported comprehension question accuracy scores for hearing L1 readers of English, deaf early signers (L2 readers of English), and hearing Chinese–English bilinguals on a sentence-reading eye-tracking task. Hearing L1 readers had the highest accuracy (92.6%), followed by deaf early signers (86.6%), then Chinese–English hearing bilinguals (83.5%). Future analyses of the SEER corpus can match readers for comprehension accuracy on the experimental eye-tracking task to test for underlying differences in reading behaviors when matched for comprehension on the experimental task as well.

Deaf participants in this sample were overall older and had more years of education compared to the hearing group. Prior studies suggest that reading skill, rather than years of education, primarily drives oculomotor differences between readers (Chamorro et al., 2017; Manly et al., 2003, 2004). Moreover, research on aging and reading has found no differences in eye-movement behaviors between young and older adults who are skilled readers (Steen-Baker et al., 2017). Given that participants in both of our groups were skilled readers, either college-educated or currently enrolled in college, and were matched on reading proficiency, it is unlikely that age or education differences account for the group effects we report. Future analyses of the SEER Corpus could more directly address this issue by matching deaf and hearing readers on age and education to further test the robustness of our findings.

Finally, evidence from the SEER Corpus provides an important opportunity to include diverse readers who engage with language in multiple modalities and assess the individual differences that contribute to successful print literacy in deaf signing bilinguals. The analyses reported here contribute to growing evidence that skilled deaf readers for whom ASL is their first and dominant language and English is their second language, experienced primarily through print, are a unique group of bilinguals. While their reading experiences could be analogous to those of hearing monolingual/English L1 readers because they primarily read and write in a single language, they split time between two functionally different languages on a daily basis. However, splitting time between two or more languages could be more analogous to the reading experiences of hearing L2 readers reading in their second language. The results presented here provide further nuance into that overlap, and it is becoming increasingly clear that either the lifelong experience of being deaf, the use of a visual gestural language, or a combination of both alters the reading process. These results and future analyses of the SEER corpus will help advance our models of reading to include more diverse readers.

## Conclusion

In this article, we present the SEER Corpus for additional analyses. The data are publicly available online (10.17605/OSF.IO/7P4F2) for other researchers to use, provided references to this article and the corpus are included in resulting scholarly work. These data will benefit researchers interested in a variety of topics, including the impact of deafness and sign language use on reading behaviors, bimodal bilingualism, and the impact of processing language exclusively in the visual modality on reading behaviors, and improving educational outcomes for deaf students. We further suggest this data will be beneficial for researchers interested more broadly in bilingualism, reading without strong reliance on speech-based codes, and overall reading efficiency to inform a more universal perspective on what it means to be a successful reader.

## Data Availability

All data and materials are available on the Open Science Framework (OSF): 10.17605/OSF.IO/7P4F2.

## Footnotes

1 The discrepancy in viewing distances between sites was an unintended oversight. However, because these differences yielded only small variation in perceived stimulus size (0.22–0.32 degrees of visual angle) and the hearing group was run on the middle viewing distance, we believe these differences are unlikely to skew the results.

## References

[CR1] Andrews, S., & Hersch, J. (2010). Lexical precision in skilled readers: Individual differences in masked neighbor priming. *Journal of Experimental Psychology: General,**139*(2), 299. 20438253 10.1037/a0018366

[CR2] Balota, D. A., Yap, M. J., Hutchison, K. A., Cortese, M. J., Kessler, B., Loftis, B., Neely, J. H., Nelson, D. L., Simpson, G. B., & Treiman, R. (2007). The English lexicon project. *Behavior Research Methods,**39*, 445–459. 17958156 10.3758/bf03193014

[CR3] Bates, D., Maechler, M., Bolker, B., Walker, S., Christensen, R. H. B., Singmann, H., ... & Bolker, M. B. (2015). Package ‘lme4’. convergence, 12(1), 2.

[CR4] Bélanger, N. N., Baum, S. R., & Mayberry, R. I. (2012a). Reading difficulties in adult deaf readers of French: Phonological codes, not guilty! *Scientific Studies of Reading,**16*(3), 263–285.

[CR5] Bélanger, N. N., Slattery, T. J., Mayberry, R. I., & Rayner, K. (2012b). Skilled deaf readers have an enhanced perceptual span in reading. *Psychological Science,**23*(7), 816–823. 10.1177/0956797611435130 22683830 10.1177/0956797611435130 PMC3723350

[CR6] Bélanger, N. N., Mayberry, R. I., & Rayner, K. (2013). Orthographic and phonological preview benefits: Parafoveal processing in skilled and less-skilled deaf readers. *Quarterly Journal of Experimental Psychology,**66*(11), 2237–2252. 10.1080/17470218.2013.780085 PMC3808502 23768045

[CR7] Bélanger, N. N., & Rayner, K. (2013). Frequency and predictability effects in eye fixations for skilled and less-skilled deaf readers. *Visual Cognition,**21*(4), 477–497. 23976872 10.1080/13506285.2013.804016 PMC3747000

[CR8] Bélanger, N. N., & Rayner, K. (2015). What eye movements reveal about deaf readers. *Current Directions in Psychological Science,**24*(3), 220–226. 10.1177/0963721414567527 26594098 10.1177/0963721414567527 PMC4651440

[CR9] Bélanger, N. N., Lee, M., & Schotter, E. R. (2018). Young skilled deaf readers have an enhanced perceptual span in reading. *The Quarterly Journal of Experimental Psychology,**71*(1), 291–301. 10.1080/17470218.2017.1324498 28447500

[CR10] Berzak, Y., & Levy, R. (2023). Eye movement traces of linguistic knowledge in native and non-native reading. *Open Mind,**7*, 179–196. 10.1162/opmi_a_00084 37416079 10.1162/opmi_a_00084 PMC10320821

[CR11] Berzak, Y., Nakamura, C., Smith, A., Weng, E., Katz, B., Flynn, S., & Levy, R. (2022). CELER: A 365-participant corpus of eye movements in L1 and L2 English reading. *Open Mind,**6*, 41–50. 10.1162/opmi_a_00054 36439073 10.1162/opmi_a_00054 PMC9692049

[CR12] Blanchard, H. E., Pollatsek, A., & Rayner, K. (1989). The acquisition of parafoveal word information in reading. *Perception & Psychophysics,**46*(1), 85–94. 2755766 10.3758/bf03208078

[CR13] Brysbaert, M., & Vitu, F. (1998). Word skipping: Implications for theories of eye movement control in reading. In Eye guidance in reading and scene perception (pp. 125–147). Elsevier Science Ltd.

[CR14] Caselli, N., Pyers, J., & Lieberman, A. M. (2021). Deaf children of hearing parents have age-level vocabulary growth when exposed to ASL by six-months. *The Journal of Pediatrics,**232*, 229. 33482219 10.1016/j.jpeds.2021.01.029 PMC8085057

[CR15] Chamorro, Y., Treviño, M., & Matute, E. (2017). Educational and cognitive predictors of pro-and antisaccadic performance. *Frontiers in Psychology,**8*, Article 2009. 29209249 10.3389/fpsyg.2017.02009 PMC5701939

[CR16] Cooley, F. G., & Quinto-Pozos, D. (2023). Examining speech-based phonological recoding during reading for adolescent deaf signers. *Journal of Experimental Psychology: General,**152*(7), 1995. 37053399 10.1037/xge0001362

[CR17] Cooley, F. G., Emmorey, K., Saunders, E., Sinclair, G., Stringer, C., & Schotter, E. R. (2025). Identifying text-based factors that contribute to the superior reading efficiency of skilled deaf readers: An eye-tracking study of length, frequency, and predictability. Journal of Experimental Psychology: Learning, Memory, and Cognition. 10.1037/xlm0001415 39928457

[CR18] Cop, U., Dirix, N., Drieghe, D., & Duyck, W. (2017). Presenting GECO: An eyetracking corpus of monolingual and bilingual sentence reading. *Behavior Research Methods,**49*(2), 602–615. 10.3758/s13428-016-0734-0 27193157 10.3758/s13428-016-0734-0

[CR19] Cop, U., Drieghe, D., & Duyck, W. (2015). Eye movement patterns in natural reading: A comparison of monolingual and bilingual reading of a novel. *PLoS One,**10*(8), Article e0134008. 10.1371/journal.pone.0134008 26287379 10.1371/journal.pone.0134008 PMC4545791

[CR20] Costello, B., Caffarra, S., Fariña, N., Duñabeitia, J. A., & Carreiras, M. (2021). Reading without phonology: ERP evidence from skilled deaf readers of Spanish. *Scientific Reports,**11*(1), Article 5202. 33664324 10.1038/s41598-021-84490-5 PMC7933439

[CR21] Dehaene, S. (2009). Reading in the brain: The new science of how we read. Viking.

[CR22] Drieghe, D., Rayner, K., & Pollatsek, A. (2008). Mislocated fixations can account for parafoveal-on-foveal effects in eye movements during reading. *Quarterly Journal of Experimental Psychology,**61*(8), 1239–1249. 10.1080/17470210701467953 10.1080/17470210701467953 PMC2662923 17853202

[CR23] Dunn, L. M., & Markwardt, F. C. (1989). *Peabody individual achievement test-revised*. American Guidance Service.

[CR24] Dye, M. W., Hauser, P. C., & Bavelier, D. (2009). Is visual selective attention in deaf individuals enhanced or deficient? The case of the useful field of view. *PLoS One,**4*(5), Article e5640. 19462009 10.1371/journal.pone.0005640 PMC2680667

[CR25] Ehrlich, S. F., & Rayner, K. (1981). Contextual effects on word perception and eye movements during reading. *Journal of Verbal Learning and Verbal Behavior,**20*(6), 641–655.

[CR26] Emmorey, K., Petrich, J. A. F., & Gollan, T. H. (2013). Bimodal bilingualism and the frequency-lag hypothesis. *Journal of Deaf Studies and Deaf Education,**18*(1), 1–11. 10.1093/deafed/ens034 23073709 10.1093/deafed/ens034 PMC3598412

[CR27] Emmorey, K., & Lee, B. (2021). The neurocognitive basis of skilled reading in prelingually and profoundly deaf adults. *Language and Linguistics Compass,**15*(2), Article e12407. 10.1111/lnc3.12407 34306178 10.1111/lnc3.12407 PMC8302003

[CR28] Emmorey, K., Akers, E., Saunders, E., Bannazadeh, M., Droubi, E., Cooley, F. G., Schotter, E. R. (2025). Assessing the effectsof sign language experience vs. deafness on the leftward reading span. *Cognitive Science.* 10.1111/cogs.70162 41449612

[CR29] Fariña, N., Duñabeitia, J. A., & Carreiras, M. (2017). Phonological and orthographic coding in deaf skilled readers. *Cognition,**168*, 27–33. 28646750 10.1016/j.cognition.2017.06.015

[CR30] Gollan, T. H., Slattery, T. J., Goldenberg, D., Van Assche, E., Duyck, W., & Rayner, K. (2011). Frequency drives lexical access in reading but not in speaking: The frequency-lag hypothesis. *Journal of Experimental Psychology: General,**140*(2), 186–209. 10.1037/a0022256 21219080 10.1037/a0022256 PMC3086969

[CR31] Gough, P. B., & Hillinger, M. L. (1980). Learning to read: An unnatural act. *Bulletin of the Orton Society,**30*, 179–196.

[CR32] Hale, J. (2001). A probabilistic Earley parser as a psycholinguistic model. In Second meeting of the North American Chapter of the Association for Computational Linguistics.

[CR33] Hall, M. L., Hall, W. C., & Caselli, N. K. (2019). Deaf children need language, not (just) speech. *First Language,**39*(4), 367–395.

[CR34] Hauser, P. C., Paludneviciene, R., Riddle, W., Kurz, K. B., Emmorey, K., & Contreras, J. (2016). American sign language comprehension test: A tool for sign language researchers. *Journal of Deaf Studies and Deaf Education,**21*(1), 64–69. 26590608 10.1093/deafed/env051

[CR35] Henner, J., Caldwell-Harris, C. L., Novogrodsky, R., & Hoffmeister, R. (2016). American sign language syntax and analogical reasoning skills are influenced by early acquisition and age of entry to signing schools for the deaf. *Frontiers in Psychology,**7*, 1982. 28082932 10.3389/fpsyg.2016.01982 PMC5183573

[CR36] Hirshorn, E. A., Dye, M. W., Hauser, P., Supalla, T. R., & Bavelier, D. (2015). The contribution of phonological knowledge, memory, and language background to reading comprehension in deaf populations. *Frontiers in Psychology,**6*, Article 1153. 26379566 10.3389/fpsyg.2015.01153 PMC4548088

[CR37] Inhoff, A. W. (1989). Parafoveal processing of words and saccade computation during eye fixations in reading. *Journal of Experimental Psychology: Human Perception and Performance,**15*(3), 544. 2527961 10.1037//0096-1523.15.3.544

[CR38] Inhoff, A. W., & Rayner, K. (1986). Parafoveal word processing during eye fixations in reading: Effects of word frequency. *Perception & Psychophysics,**40*(6), 431–439. 3808910 10.3758/bf03208203

[CR39] Kaufman, A. S., & Kaufman, N. L. (2004). Kaufman brief intelligence test. *Journal of Psychoeducational Assessment,**28*(2), 167–174. 10.1177/0734282909348217

[CR40] Kaye, N. G., & Gordon, P. C. (2025). Sentence processing by humans and machines: Large language models as a tool to better understand human reading. Psychonomic Bulletin & Review. 10.3758/s13423-025-02756-9 10.3758/s13423-025-02756-9 40804188

[CR41] Kliegl, R., Grabner, E., Rolfs, M., & Engbert, R. (2004). Length frequency and predictability effects of words on eye movements in reading. European Journal of Cognitive Psychology, 16(1-2), 262–284 10.1080/09541440340000213

[CR42] Kliegl, R., Nuthmann, A., & Engbert, R. (2006). Tracking the mind during reading: the influence of past, present, and future words on fixation durations. J*ournal of experimental psychology: General, 135*(1), 12. 10.1037/0096-3445.135.1.12 16478314

[CR43] Bellugi, U., & Klima, E. (1979). Language: Perspectives from another modality. *Brain and Mind,**69*, 99–117. 10.1002/9780470720523.ch6 261658

[CR44] Kuperman, V., Siegelman, N., Schroeder, S., Acartürk, C., Alexeeva, S., Amenta, S., Bertram, R., Bonandrini, R., Brysbaert, M., Chernova, D., Da Fonseca, S. M., Dirix, N., Duyck, W., Fella, A., Frost, R., Gattei, C. A., Kalaitzi, A., Lõo, K., Marelli, M., … Usal, K. A. (2023). Text reading in English as a second language: Evidence from the Multilingual EyeMovements Corpus. *Studies in Second Language Acquisition,**45*(1), 3–37. 10.1017/S0272263121000954

[CR45] Kuperman, V., Schroeder, S., Acartürk, C., Agrawal, N., Bollinger, L., Brasser, J., ... & Siegelman, N. (2024). New data on text reading in English as a second language: the wave 2 expansion of the multilingual eye movements corpus (MECO). Studies in Second Language Acquisition.

[CR46] Levy, R. (2008). Expectation-based syntactic comprehension. *Cognition,**106*(3), 1126–1177. 17662975 10.1016/j.cognition.2007.05.006

[CR47] Liddell, S. K. (2003). *Grammar, gesture, and meaning in American Sign Language*. Cambridge University Press.

[CR48] Lu, X., & Ai, H. (2015). Syntactic complexity in college-level English writing: Differences among writers with diverse L1 backgrounds. *Journal of Second Language Writing,**29*, 16–27.

[CR49] Liu, Z. F., Chen, C. Y., Tong, W., & Su, Y. Q. (2021). Deafness enhances perceptual span size in Chinese reading: Evidence from a gaze‐contingent moving‐window paradigm abstract. *PsyCh Journal 10*(4) 508–520. 10.1002/pchj.v10.410.1002/pchj.442 10.1002/pchj.442 33899345

[CR50] Luke, S. G., & Christianson, K. (2018). The Provo corpus: A large eye-tracking corpus with predictability norms. *Behavior Research Methods,**50*, 826–833. 28523601 10.3758/s13428-017-0908-4

[CR51] Manly, J. J., Touradji, P., Tang, M. X., & Stern, Y. (2003). Literacy and memory decline among ethnically diverse elders. *Journal of Clinical and Experimental Neuropsychology,**25*(5), 680–690. 12815505 10.1076/jcen.25.5.680.14579

[CR52] Manly, J. J., Byrd, D. A., Touradji, P., & Stern, Y. (2004). Acculturation, reading level, and neuropsychological test performance among African American elders. *Applied Neuropsychology,**11*(1), 37–46. 15471745 10.1207/s15324826an1101_5

[CR53] Mayberry, R. I., Del Giudice, A. A., & Lieberman, A. M. (2011). Reading achievement in relation to phonological coding and awareness in deaf readers: A meta-analysis. *Journal of Deaf Studies and Deaf Education,**16*(2), 164–188. 21071623 10.1093/deafed/enq049 PMC3739043

[CR54] McConkie, G. W., & Rayner, K. (1975). The span of the effective stimulus during a fixation in reading. *Perception & Psychophysics,**17*(6), 578–586.

[CR55] Nahatame, S., Ogiso, T., Kimura, Y., & Ushiro, Y. (2024). TECO: An eye-tracking corpus of Japanese L2 English learners’ text reading. *Research Methods in Applied Linguistics,**3*(2), Article 100123. 10.1016/j.rmal.2024.100123

[CR56] Radford, A., Wu, J., Child, R., Luan, D., Amodei, D., & Sutskever, I. (2019). Language models are unsupervised multitask learners. OpenAI blog, 1(8), 9. https://www.persagen.com/files/misc/radford2019language.pdf

[CR57] Rayner, K. (1998). Eye movements in reading and information processing: 20 years of research. *Psychological Bulletin,**124*(3), 372. 9849112 10.1037/0033-2909.124.3.372

[CR58] Rayner, K. (2009). Eye movements in reading: Models and data. *Journal of Eye Movement Research,**2*(5), Article 1. 20664810 PMC2906818

[CR59] Rayner, K. (2014). The gaze-contingent moving window in reading: Development and review. *Visual Cognition,**22*(3–4), 242–258.

[CR60] Rayner, K., & Liversedge, S. P. (2011). Linguistic and cognitive influences on eye movements during reading.

[CR61] Rayner, K., & McConkie, G. W. (1976). What guides a reader’s eye movements? *Vision Research,**16*(8), 829–837. 960610 10.1016/0042-6989(76)90143-7

[CR62] Rayner, K., Slattery, T. J., Drieghe, D., & Liversedge, S. P. (2011). Eye movements and word skipping during reading: Effects of word length and predictability. *Journal of Experimental Psychology: Human Perception and Performance,**37*(2), 514. 21463086 10.1037/a0020990 PMC3543826

[CR63] Schotter, E. R., & Dillon, B. (2025). A beginner’s guide to eye tracking for psycholinguistic studies of reading. *Behavior Research Methods,**57*(2), Article 68. 39843882 10.3758/s13428-024-02572-4

[CR64] Schotter, E. R., Stringer, C., Saunders, E., Cooley, F. G., Sinclair, G., & Emmorey, K. (2024). The role of perceptual and word identification spans in reading efficiency: Evidence from hearing and deaf readers. *Journal of Experimental Psychology: General,**153*(10), 2359. 39388114 10.1037/xge0001633

[CR65] Schotter, E. R., Angele, B., & Rayner, K. (2012). Parafoveal processing in reading. *Attention, Perception, & Psychophysics,**74*, 5–35. 10.3758/s13414-011-0219-2 22042596

[CR66] Sehyr, Z. S., & Emmorey, K. (2022). Contribution of lexical quality and sign language variables to reading comprehension. *The Journal of Deaf Studies and Deaf Education,**27*(4), 355–372. 10.1093/deafed/enac018 35775152 10.1093/deafed/enac018

[CR67] Siegelman, N., Schroeder, S., Acartürk, C., Ahn, H. D., Alexeeva, S., Amenta, S., & Kuperman, V. (2022). Expanding horizons of cross-linguistic research on reading: The multilingual eye-movement corpus (MECO). *Behavior Research Methods,**54*(6), 2843–2863. 35112286 10.3758/s13428-021-01772-6 PMC8809631

[CR68] Sui, L., Dirix, N., Woumans, E., & Duyck, W. (2022). GECO-CN: Ghent Eye-tracking COrpus of sentence reading for Chinese-English bilinguals. *Behavior Research Methods*. 10.3758/s13428-022-01931-3 35896891 10.3758/s13428-022-01931-3

[CR69] Staub, A. (2015). The effect of lexical predictability on eye movements in reading: Critical review and theoretical interpretation. *Language and Linguistics Compass,**9*(8), 311–327.

[CR70] Staub, A., & Rayner, K. (2007). Eye movements and on-line comprehension processes. *The Oxford Handbook of Psycholinguistics,**327*, 342.

[CR71] Steen-Baker, A. A., Ng, S., Payne, B. R., Anderson, C. J., Federmeier, K. D., & Stine-Morrow, E. A. (2017). The effects of context on processing words during sentence reading among adults varying in age and literacy skill. *Psychology and Aging,**32*(5), 460. 28816473 10.1037/pag0000184

[CR72] Supalla, T., & Newport, E. (1978). How many seats in a chair? In P. Siple (Ed.), *Understanding language through sign language research* (pp. 158–181). Academic Press.

[CR73] Stringer, C., Cooley, F., Saunders, E., Emmorey, K., & Schotter, E. R. (2024). Deaf readers use leftward information to read more efficiently: Evidence from eye tracking. *Quarterly Journal of Experimental Psychology,**77*(10), 2098–2110. 10.1177/17470218241232407 10.1177/17470218241232407 38326329

[CR74] Taylor, W. L. (1956). Recent developments in the use of “Cloze Procedure.” *Journalism Quarterly,**33*(1), 42–99.

[CR75] Thierfelder, P., Wigglesworth, G., & Tang, G. (2020). Orthographic and phonological activation in Hong Kong deaf readers: An eye-tracking study. *Quarterly Journal of Experimental Psychology,**73*(12), 2217–2235. 10.1177/1747021820940223 10.1177/1747021820940223 32564689

[CR76] Traxler, M. J., Banh, T., Craft, M. M., Winsler, K., Brothers, T. A., Hoversten, L. J., Piñar, P., & Corina, D. P. (2021). Word skipping in deaf and hearing bilinguals: Cognitive control over eye movements remains with increased perceptual span. *Applied Psycholinguistics,**42*(3), 601–630. 10.1017/S0142716420000740

[CR77] Valli, C., Lucas, C., Mulrooney, K. J., & Villanueva, M. (2011). *Linguistics of American Sign Language* (5th ed.). Gallaudet University Press.

[CR78] Whitford, V., & Titone, D. (2012). Second-language experience modulates first-and second-language word frequency effects: Evidence from eye movement measures of natural paragraph reading. *Psychonomic Bulletin & Review,**19*(1), 73–80. 22042632 10.3758/s13423-011-0179-5

[CR79] Wolf, M. (2007). Proust and the squid: The story and science of the reading brain. New York.

[CR80] Wong, R., Veldre, A., & Andrews, S. (2024). Are there independent effects of constraint and predictability on eye movements during reading? *Journal of Experimental Psychology: Learning, Memory, and Cognition,**50*(2), 331. 36521159 10.1037/xlm0001206

[CR81] Yan, G., Lan, Z., Meng, Z., Wang, Y., & Benson, V. (2021). Phonological coding during sentence reading in Chinese deaf readers: An eye-tracking study. *Scientific Studies of Reading,**25*(4), 287–303. 10.1080/10888438.2020.1778000

[CR82] Yan, M., Pan, J., Bélanger, N. N., & Shu, H. (2015). Chinese deaf readers have early access to parafoveal semantics. *Journal of Experimental Psychology: Learning, Memory, and Cognition,**41*(1), 254–261. 10.1037/xlm000003 24999711 10.1037/xlm0000035 PMC4286537

